# Correlation between synthetic MRI relaxometry and apparent diffusion coefficient in breast cancer subtypes with different neoadjuvant therapy response

**DOI:** 10.1186/s13244-023-01492-9

**Published:** 2023-09-29

**Authors:** Wenhong Jiang, Siyao Du, Si Gao, Lizhi Xie, Zichuan Xie, Mengfan Wang, Can Peng, Jing Shi, Lina Zhang

**Affiliations:** 1 Department of Radiology, The First Hospital of China Medical University, Shenyang, China; 2 GE Healthcare, MR Research China, Beijing, China; 3 Guangzhou institute of technology, Xidian University, Guangzhou, China; 4 Department of Medical Oncology, The First Hospital of China Medical University, Shenyang, China

**Keywords:** Synthetic magnetic resonance imaging, Apparent diffusion coefficient, Breast cancer, Neoadjuvant therapy

## Abstract

**Background:**

To evaluate the correlation between synthetic MRI (syMRI) relaxometry and apparent diffusion coefficient (ADC) maps in different breast cancer subtypes and treatment response subgroups.

**Methods:**

Two hundred sixty-three neoadjuvant therapy (NAT)-treated breast cancer patients with baseline MRI were enrolled. Tumor annotations were obtained by drawing regions of interest (ROIs) along the lesion on T1/T2/PD and ADC maps respectively. Histogram features from T1/T2/PD and ADC maps were respectively calculated, and the correlation between each pair of identical features was analyzed. Meanwhile, features between different NAT treatment response groups were compared, and their discriminatory power was evaluated.

**Results:**

Among all patients, 20 out of 27 pairs of features weakly correlated (*r* = – 0.13–0.30). For triple-negative breast cancer (TNBC), features from PD map in the pathological complete response (pCR) group (*r* = 0.60–0.86) showed higher correlation with ADC than that of the non-pCR group (*r* = 0.30–0.43), and the mean from the ADC and PD maps in the pCR group strongly correlated (*r* = 0.86). For HER2-positive, few correlations were found both in the pCR and non-pCR groups. For luminal HER2-negative, T2 map correlated more with ADC than T1 and PD maps. Significant differences were seen in T2 low percentiles and median in the luminal-HER2 negative subtype, yielding moderate AUCs (0.68/0.72/0.71).

**Conclusions:**

The relationship between ADC and PD maps in TNBC may indicate different NAT responses. The no-to-weak correlation between the ADC and syMRI suggests their complementary roles in tumor microenvironment evaluation.

**Critical relevance statement:**

The relationship between ADC and PD maps in TNBC may indicate different NAT responses, and the no-to-weak correlation between the ADC and syMRI suggests their complementary roles in tumor microenvironment evaluation.

**Key points:**

• The relationship between ADC and PD in TNBC indicates different NAT responses.

• The no-to-weak correlations between ADC and syMRI complementarily evaluate tumor microenvironment.

• T2 low percentiles and median predict NAT response in luminal-HER2-negative subtype.

**Graphical Abstract:**

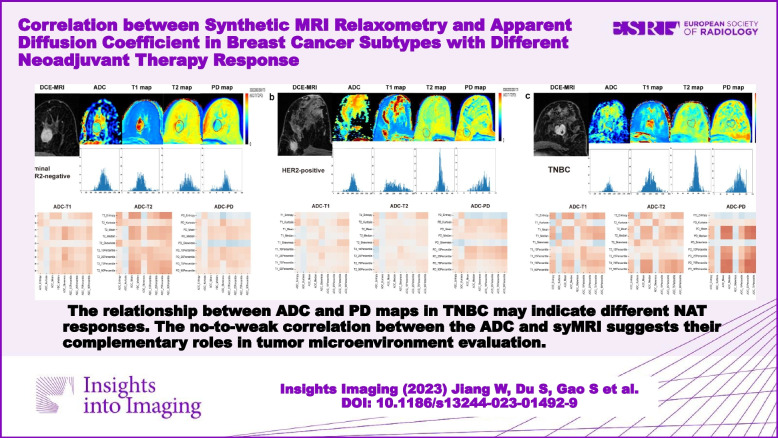

## Introduction

Breast cancer is the most common malignancy and the second leading cause of tumor-related mortality in women worldwide [1]. Neoadjuvant therapy (NAT) has been established as a standard treatment for most breast cancers, especially locally advanced breast cancer (LABC) [2]. A pathologic complete response (pCR) following NAT would indicate a very promising disease-free and overall survival rate [3]. However, treatment responses vary among patients, and approximately 2–30% do not benefit from NAT [4]. Those non-responders receiving NAT would miss the optimal timing of surgery and suffer from unnecessary side effects. Thus, the prediction of therapeutic efficacy in advance could be conducive to the optimal selection of the overall treatment protocol [5, 6].

Magnetic resonance imaging (MRI) is a preferred modality for monitoring treatment response as it offers both functional and morphological information [7–9], and it has proven clinical utility in neoadjuvant therapy efficacy prediction [10–12]. Besides regular sequences, diffusion-weighted imaging (DWI) as a functional imaging method can be quantified by the apparent diffusion coefficient (ADC), which reflects cellularity and interstitial water mobility. Several studies have investigated the additive value of ADC in the prediction of treatment response to NAT in breast cancer patients [10, 13, 14]. However, many research results vary due to the different scanners and sequence parameters [15–17].

The recently proposed synthetic MRI (syMRI) [18–20] uses a multiple-dynamic multiple-echo (MDME) acquisition method for simultaneous measurement of quantitative values including T1 and T2 relaxation time and proton-density (PD) mapping in one single scan, with the advantage of shorter scan time and independence of MRI scanners and parameters [21, 22]. Several studies have confirmed the clinical utility of syMRI in malignancy identification [23], molecular typing [24], and therapeutic response assessment [25, 26] in various cancers. ADC has been the most common sequence in combination with syMRI for differential diagnosis [27, 28], prognostic prediction [29], and NAT response evaluation [30]. Our earlier work has substantiated that T1 relaxation time combined with ADC effectively predicted pathological response after one NAT cycle in breast cancer [30]. However, there is limited literature on the correlation between syMRI and ADC, particularly among the various breast cancer subtypes. It remains unclear how tumor heterogeneity determines the complexity of the relationship between syMRI and ADC. This study aimed to explore the heterogeneity and correlation of parametric maps from T1/T2/PD and ADC mapping in different LABC subtypes with different NAT responses, trying to provide a basis for future research on underlying biological mechanisms.

## Materials and methods

The institutional review board in our hospital approved this retrospective study and waived the requirement for informed consent.

### Patient population

We initially recruited 284 LABC patients who performed pretreatment breast MRI from March 2019 to August 2022. The eligibility criteria were as follows: (1) pathological confirmation of primary breast cancer and scheduled NAT before surgery; (2) no history of treatment for breast cancer; (3) complete pathological grading and immunohistochemical (IHC) receptor status information; and (4) baseline breast MRI including syMRI, DWI, and dynamic contrast enhanced (DCE)-MRI sequences. Of 284 patients, 21 were excluded for the following reasons: (1) tumor diameter on DCE-MRI less than 1.0 cm (*n* = 6); (2) insufficient MRI image quality to obtain measurements (*n* = 4); (3) incomplete standard NAT cycles (*n* = 8); and (4) surgery having been performed at an outside institution (*n* = 3). Finally, 263 patients (mean age 51 ± 11 years, age range 24–75 years) were enrolled for further analysis.

### Histopathological analysis

Histologic grade, estrogen receptor (ER), progesterone receptor (PR), and human epidermal growth factor receptor 2 (HER2) were evaluated from the histopathologic reports of ultrasound-guided core biopsies performed before NAT. The positivity for ER, PR, and HER2 was defined according to the American Society of Clinical Oncology (ASCO)/College of American Pathologists (CAP) guidelines, as summarized in Supplemental Table 1 [31, 32]. The Ki-67 index was assessed with a cut-off value of 20% [33]. The molecular subtypes were stratified into luminal HER2-negative (ER- and/or PR-positive and HER2-negative), HER2-positive (HER2-positive regardless of HR status), and triple-negative breast cancer (TNBC, ER-, PR- and HER2-negative) subgroups based on biopsy specimen analysis.

**Table 1:**
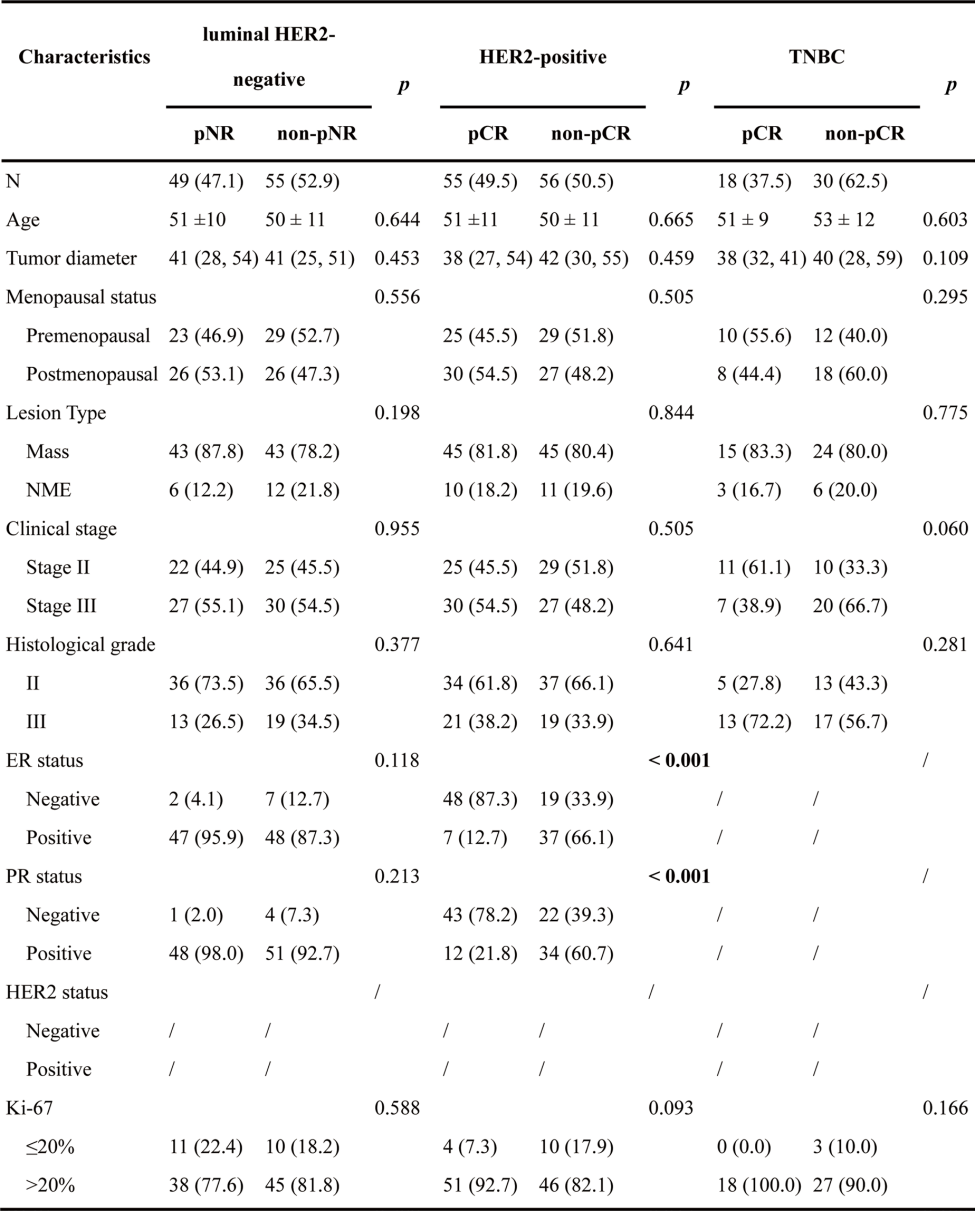
Clinicopathological characteristics

Bolded text with *p* values indicates statistical significance. Age is presented as mean ± SD. Tumor diameter is presented as median (interquartile range), and the others are shown as proportions (percentages)

*pCR* Pathologic complete response, *pNR*, Pathologic non-response, *ER* Estrogen receptor, *PR* Progesterone receptor, *HER2* Human epidermal growth factor receptor 2, *TNBC* Triple-negative breast cancer, *NME* Non-mass enhancement

The pathological response after NAT was assessed through the examination of the tumor bed after surgery using the Miller-Payne (MP) grading system. pCR was defined as the absence of residual invasive cancer in the surgical specimen (ductal carcinoma in situ could be present), as in MP grade 5. MP grades 1–2 indicate pathological non-response (pNR). Regarding clinical practice, the response of the luminal HER2-negative subtype was dichotomized as pNR and non-pNR given its low pCR rates, while the HER2-positive and TNBC subtypes used dichotomous pCR and non-pCR [34].

### Synthetic relaxation and ADC mapping acquisition

The MRI scanning protocol was described in detail in our previous study [30]. MRI examinations were performed using a 3-T MRI scanner (Signa Pioneer, GE Healthcare, Milwaukee, USA) with a dedicated 8-channel bilateral breast coil with the patient in the prone position. The scan sequences included axial fast spin-echo (FSE) T1WI and fat-suppression (FS) T2WI, syMRI, DWI (*b* = 0, 50, 400, 800 s/mm^2^), and differential subsampling with cartesian ordering (DISCO) DCE-MRI. SyMRI used a 2D FSE MDME sequence (scan time: 3: 09 min) before contrast agent injection, with the following parameters: TR = 5600 ms, TE = 22.1/110.4 ms, TI = NA, field of view = 360 × 360 mm, matrix = 192 × 180, section thickness = 5 mm, intersection gap = 1.3 mm, number of sections = 25, and acceleration factor = 2.5. Details of the other MRI sequences are presented in Supplemental Table 2.

**Table 2:**
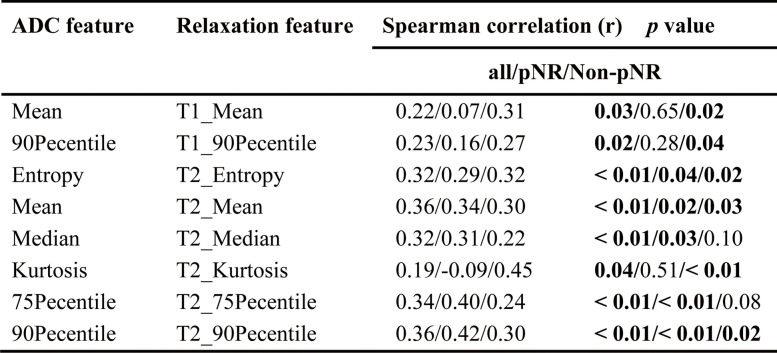
Spearman correlation analysis of ADC and relaxation features in the luminal-HER2 negative subtype

Bolded text with *p* values indicates statistical significance

*pNR* Pathologic non-response, *ADC* Apparent diffusion coefficient, *HER2* Human epidermal growth factor receptor 2

### Image analysis and feature extraction

The MAGiC software was used to import the syMRI sequence to yield T1, T2, and PD quantitative maps for measurements and produce synthetic images matching conventional images. The ReadyView software (GE Healthcare, Milwaukee, Wisconsin) was used to process the DWI data. The following equation was used to calculate ADC maps: ADC = − ln [S0/S1]/b1, where S0 and S1 are the signal intensities in the ROIs determined by two gradient factors and b1 is 800 s/mm^2^.

With reference to DCE-MRI, regions of interest (ROIs) of tumor were manually segmented along the tumor margin slice-by-slice by Reader A (with 5 years of experience in breast imaging) and then reviewed by Reader B (with 10 years of experience in breast imaging) using ITK-SNAP (version 3.8.0, http://www.itksnap.org). The two readers were aware that the patients were diagnosed with breast cancer but blinded to biopsy biomarkers and treatment outcomes after NAT. The first and last slices of tumors were excluded to eliminate partial volume effects. In the syMRI, ROIs were manually drawn on synthetic T2-weighted images and were automatically mapped to other relaxation maps. In the DWI, ROIs of the same lesion were drawn on the DWI *b* = 800 and copied to ADC images with manual alignment. Necrotic, hemorrhagic, and cystic components were included in the ROIs. The largest tumor was selected as the index tumor when there were multiple lesions. Tumor size was measured as the maximum diameter at the maximum cross-section of a transverse DCE-MRI.

Feature extraction was performed with Python (version 3.7.0, http://www.python.org). For each tumor, nine histogram features were extracted from T1/T2/PD and ADC maps, including the mean, median, entropy, kurtosis, skewness, and the 10/25/75/90th percentiles (their definitions and interpretations are presented in Supplemental Table 3).

**Table 3:**
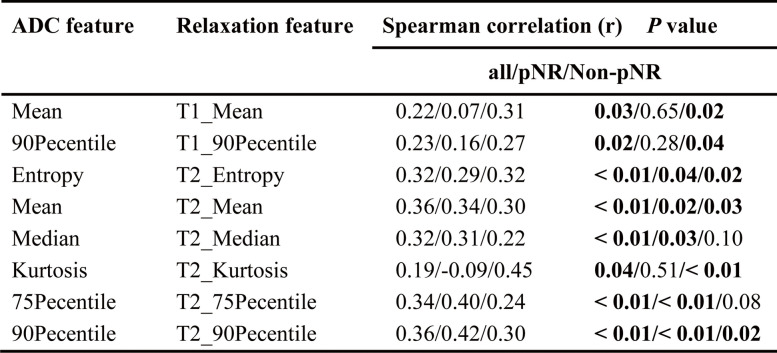
Spearman correlation analysis of ADC and relaxation features in the TNBC subtype

Bolded text with *p* values indicates statistical significance

*pCR* Pathologic complete response, *ADC* Apparent diffusion coefficient, *TNBC* Triple-negative breast cancer, *PD* Proton density

### Statistical analysis

All statistical analysis was performed with R software (version 3.6.1, http://www.r-project.org/) and EmpowerStats (http://www.empowerstats.com, X & Y Solutions, Inc.). After excluding symmetrical duplicates in intra-sequence scenarios, Spearman’s rank correlation coefficients were calculated among each pair of identical histogram features from the ADC and T1/T2/PD maps. Correlation coefficients below 0.49 were said to show weak correlation, 0.50 to 0.79 moderate correlation, and 0.80 to 1.0 strong correlation [35]. Finally, the Mann-Whitney *U* test and Student’s *t*-test were performed to compare differences between the features for different subtypes. The receiver operating characteristic curve (ROC) and the area under the curve (AUC) were used to assess the performance of the imaging features of various subtypes in discriminating therapy response. For all analyses performed, the significance threshold was set to 0.05.

## Results

### Patient characteristics

**Fig. 1:**
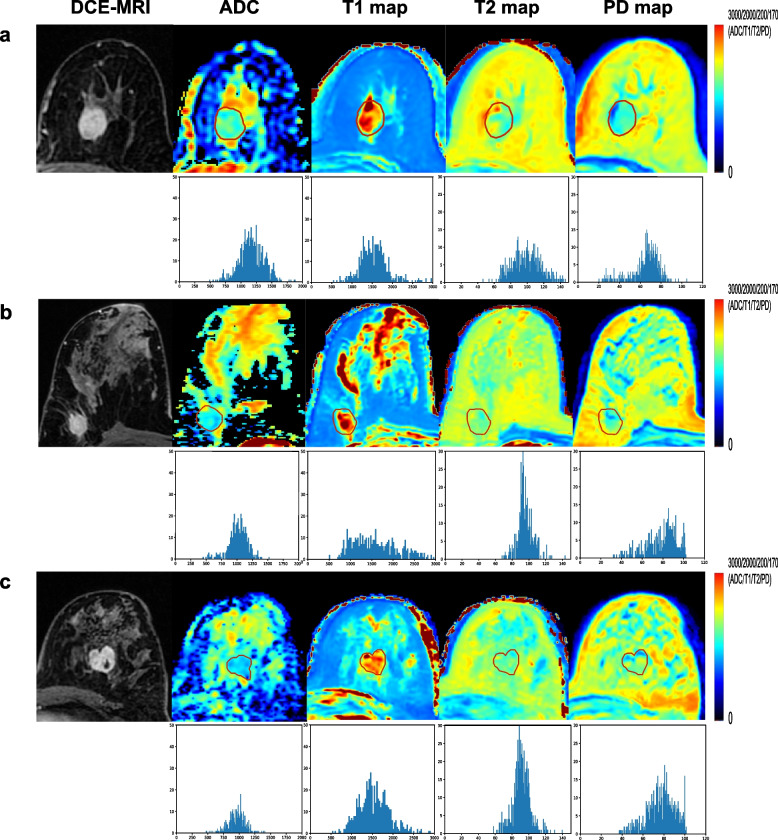
Representative patients’ images and histograms in three subtypes. **a** Luminal HER2-negative: 56-year-old patient. **b** HER2-positive: 63-year-old patient. **c** TNBC: 46-year-old patient. DCE, dynamic contrast enhancement; ADC, apparent diffusion coefficient; HER2, human epidermal growth factor receptor 2; TNBC, triple-negative breast cancer; PD, proton density

Among the 263 enrolled patients, 104 (39.5%) were luminal HER2-negative, 111 (42.2%) were HER2-positive, and 48 (18.3%) were TNBC. According to postoperative pathological results, 49 (47.1%) patients were classified as pNR and 55 (52.9%) as non-pNR in the luminal HER2-negative subtype, and the pCR rates were 49.5% (55 of 111) and 37.5% (18 of 48) in the HER2-positive and TNBC subtypes, respectively. The MRI morphological features and clinicopathological characteristics are summarized in Table 1. The HER2-positive subtype differed significantly in ER and PR status between the non-pCR and pCR groups. Representative patients’ images and histograms are shown in Fig. 1.

### Features correlation in the whole population

**Fig. 2:**
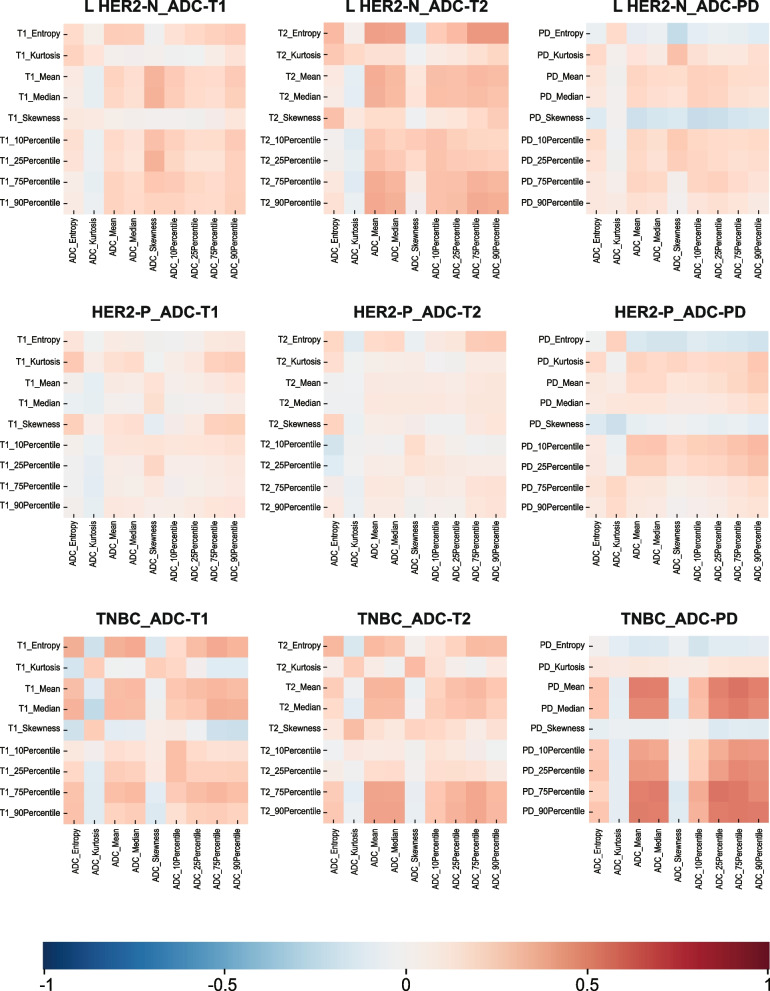
Correlation matrix for all image-derived features in various subtypes. Several features demonstrated a high correlation, particularly the features from the ADC and T2 maps in the luminal HER2-negative subtype and from the ADC and PD maps in the TNBC subtype. HER2, human epidermal growth factor receptor 2; L HER2-N, luminal HER2-negative; HER2-P, HER2-positive; TNBC, triple-negative breast cancer; ADC, apparent diffusion coefficient; PD, proton density

No strong or moderate correlation was observed in the whole population. 20 out of 27 pairs of features presented a fairly weak correlation (*r* = − 0.13–0.30,* p* < 0.05), with the entropy from the ADC and T2 maps showing the highest correlation (*r* = 0.30, *p* < 0.01). Figure 2 depicts the correlogram corresponding to the cross-correlation matrix for each subtype’s histogram features. Additional correlation coefficients and *p* values between ADC and T1/T2/PD maps in the whole population and each subtype are shown in Supplemental Table 4.

**Table 4:**
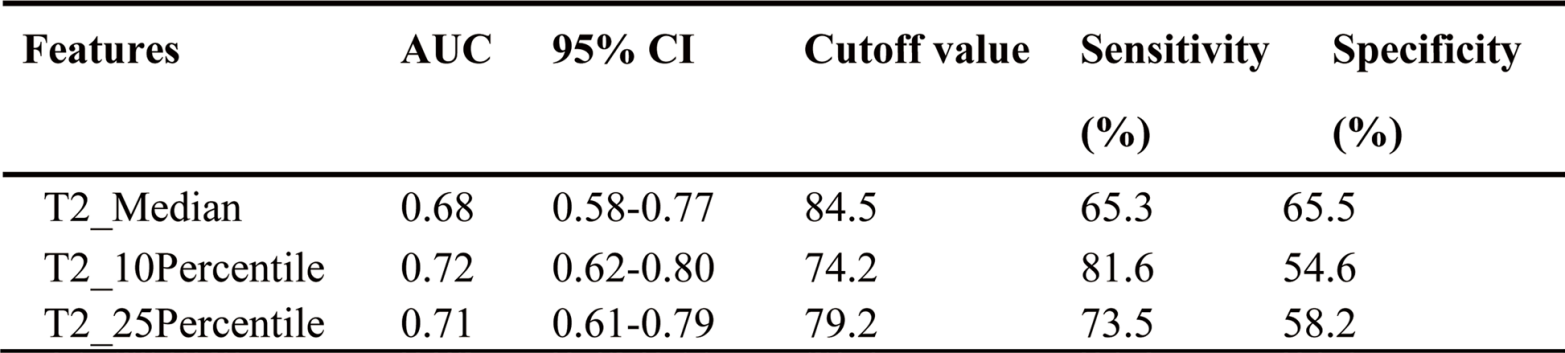
Diagnostic performance of features of significant difference in luminal HER2-negative subtype

Here indicates the diagnostic performance of discriminating pNR and non-pNR

*AUC* Area under the receiver operating characteristic curve, *CI* Confidence interval, *pNR* Pathologic non-response

### Features correlation in the luminal HER2-negative subtype

In the luminal HER2-negative subtype, the overall correlation was slightly higher than that of the whole population, and features from the T2 map (*r* = 0.19–0.36, *p* < 0.05) correlated more with ADC map than those from the T1 and PD maps (*r* = 0.21–0.23, *p* < 0.05).

No strong or moderate correlation was observed in either the pNR or non-pNR groups. Weak correlations were observed in the mean and 90th percentile from the ADC and T1 maps and entropy, mean, kurtosis, and 90th percentile from the ADC and T2 maps in the non-pNR group (*r* = 0.27–0.45, *p* < 0.05). However, in the pNR group, only the entropy, mean, median, and 75/90th percentiles from the ADC and T2 maps demonstrated a weak correlation (*r* = 0.29–0.42, *p* < 0.05) (Table 2).

### Features correlation in the HER2-positive subtype

In the HER2-positive subtype, the entropy from the ADC and T2 maps and the 10/25th percentiles from the ADC and PD maps weakly correlated (*r* = 0.20–0.24, *p* < 0.05).

No strong or moderate correlation was observed in either the pCR or non-pCR groups. Weak correlations were observed only in the skewness from the ADC and T1 maps (*r* = − 0.27, *p* = 0.04) in the pCR group.

### Feature correlation in the TNBC subtype

In the TNBC subtype, the entropy, mean, median, and 10/75th percentiles from the ADC and T1 maps (*r* = 0.30–0.36, *p* < 0.05), the entropy, mean, median, and 75/90th percentiles from the ADC and T2 maps (*r* = 0.31–0.39, *p* < 0.05), and the mean, median, and 25/75/90th percentiles from the ADC and PD maps (*r* = 0.41–0.53, *p* < 0.05) showed positive correlation.

In the pCR group, strong correlations were observed in the mean (*r* = 0.86, *p* < 0.01) from the ADC and PD maps. Moderate correlations were observed in skewness from the ADC and T1 maps (*r* = 0.51, *p* = 0.03), skewness from the ADC and T2 maps (*r* = 0.51, *p* = 0.03), and the median and 25/75/90th percentiles from the ADC and PD maps (*r* = 0.78/0.65/0.70/0.60, *p* < 0.05) in the pCR group as well (Table 3). Weak correlation coefficients and *p* values between ADC and T1/T2/PD maps are shown in Supplemental Table 4.

### Significant features for NAT response prediction in various subtypes

Significant differences were seen in the median and 10/25th percentiles (AUC = 0.68/0.72/0.71) from the T2 map between pNR and non-pNR in the luminal-HER2 negative subtype. Table 4 shows the diagnostic performance of these features. No significant differences were noted in the HER2-positive and TNBC subtypes, as well as ADC, T1, and PD maps.

## Discussion

Our study demonstrated no-to-strong correlation between T1/T2/PD and ADC maps of LABC, which differed in various molecular subtypes and treatment response groups. ADC map of the HER2-positive subtype generally had a poor correlation with all maps in both pCR and non-pCR groups. Paired PD and ADC features in the pCR group of the TNBC subtype highly correlated, but this was not the case for the non-pCR group. The features from the T2 map correlated more with ADC map than those from the T1 and PD maps in the luminal HER2-negative subtype.

The mean and percentiles showed generally positive correlation between the T1/T2/PD and ADC maps in all patients. This suggests that a high cell density and nuclear-cytoplasmic ratio in malignancies lead to a corresponding decrease in extracellular space and free water content, thus T1/T2/PD and ADC features decrease in synchrony. In addition, tumor heterogeneity resulted in weak correlations in all patient analyses. Considering the effect of molecular subtypes, a stratified analysis was performed and the correlation results were diverse.

In the HER2-positive group, few significant correlations were found. Previous studies [36, 37] have found that HER2-positive tumors exhibit an increased ADC value compared with HER2-negative tumors, which suggests that HER2-positive tumors experience more angiogenesis, while T1/T2 does not increase significantly [24]. Although all maps assess water molecules, the T1/T2 relaxation times are particularly sensitive to edema, water (hydrogen) exchange on ionizable groups across membranes, as well as macromolecular hydration layers relative to bulk water [38]. The ADC is sensitive to the translational diffusion of water in tissue, which is affected by the degree of water translational restriction that occurs primarily by lipids or lipid bilayers [39]. Hence, T1/T2 and ADC are often combined to determine benign and malignant lesions or therapeutic efficacy in multivariate analysis [27, 28, 40], implying that their relationships appear complementary rather than duplicative. The weak or no correlation between the T1/T2 and ADC maps suggests that they reveal tumor water molecular characteristics in fundamentally different ways, thus can indeed be used as complementary contrast-free techniques to evaluate the tumor microenvironment.

In the TNBC, the mean and multiple percentiles from the PD and ADC maps showed moderate-to-strong correlations only for the pCR group. Effective PD evaluation was lacking in tumor imaging due to the low contrast between tumor and normal tissue. A recent syMRI study [27] found that the PD and ADC of malignant tumors are significantly lower than those of benign lesions, and in the present study, we further explored the correlation between PD and ADC maps. The abundance of protons is one of the most important factors that affect the diffusion coefficient. The tumors of the pCR subgroup in TNBC tend to have a small size, uniform signal, and homogeneous enhancement, making PD and ADC highly consistent [41]. TNBC without pCR is more prone to intratumoral heterogeneity from tumor ischemia and necrosis, hemorrhage, and edema, thereby weakening the correlation between PD and ADC [24, 42]. Interestingly, no strong correlation was found between PD and ADC in the HER2-positive and luminal HER2-negative subtypes. We speculate that such inconsistency is possibly due to the abundant immature neovascularization of the HER2-positive subtype and the low proliferation of the luminal HER2-negative subtype. Though our results may need clinical validation with larger sample sizes, the strong correlation between PD and ADC maps is expected to be an effective contrast-free technique to predict the chemosensitivity of TNBC.

Note that the pNR and non-pNR subgroupings were used in the luminal HER2-negative subtype due to its low pCR rate. For this subtype, the correlations of most features between ADC and T2 were slightly higher than in the whole population. Due to their low intrinsic proliferation, patients with pNR are insensitive to chemotherapy, presenting with loose connective tissue and sparsely scattered tumor cells. This may explain the increased correlation between T2 and ADC for this subtype.

Additionally, the low percentiles and median of T2 in the luminal-HER2-negative subtype at baseline were independent predictors of pNR. Unlike our study, Matsuda et al. [26] found that the SD of T2 from syMRI could predict pCR with an AUC of 0.829. This inconsistency is probably due to differences in research designs and sample sizes. Our study included a larger cohort and subtype analysis. We also found that baseline ADC cannot predict treatment response, which agrees with previous studies [15, 16]. Longitudinal monitoring is usually necessary for T1/T2 and ADC prediction. Extra habitat analysis utilizing pixel clustering of T1/T2/PD and ADC may open new opportunities for noninvasive assessment of tumor heterogeneity and drug resistance.

## Limitations

This study suffers from several limitations. First, evaluation based only on imaging did not allow us to determine pathophysiological origins. Future research should explore the molecular mechanisms underlying such effects. Second, correlation analysis was performed at the feature level rather than the voxel level, and future voxel-voxel analyses are needed to validate our results. Third, the stratified analysis resulted in smaller numbers of each subtype, and larger cohorts and survival data are desired to confirm and extend the results of this study.

## Conclusion

The no-to-weak correlation between the ADC and relaxation maps suggests their complementary roles in tumor microenvironment evaluation. The relationship between ADC and PD maps in the TNBC may indicate different NAT responses. However, their physiological significance needs to be further explored.

## Data Availability

The datasets used and analyzed during the current study are available from the corresponding author on reasonable request.

## Abbreviations

ADC: Apparent diffusion coefficient
AUC: Area under ROC curve
DCE: Dynamic contrast enhanced
DISCO: Differential subsampling with cartesian ordering
DWI: Diffusion-weighted imaging
ER: Estrogen receptor
HER2: Human epidermal growth factor receptor-2
IHC: Immunohistochemical
LABC: Locally advanced breast cancer
MDME: Multiple-dynamic multiple-echo
MP: Miller-Payne
MRI: Magnetic resonance imaging
NAT: Neoadjuvant therapy
pCR: Pathological complete response
PD: Proton density
pNR: Pathological non-response
PR: Progesterone receptor
ROC: Receiver operating characteristic curve
ROIs: Regions of interest
TNBC: Triple-negative breast cancer
